# Dermolipoma in an Unusual Location: A Case Report and Literature Review

**DOI:** 10.7759/cureus.39241

**Published:** 2023-05-19

**Authors:** Steffani Krista Someda, Yasuhiro Takahashi

**Affiliations:** 1 Oculoplastic, Orbital and Lacrimal Surgery, Aichi Medical University Hospital, Aichi, JPN

**Keywords:** cosmesis, complete excision, computed tomography, inferonasal, dermolipoma

## Abstract

A healthy nine-year-old Japanese male presented with a history of inferonasal conjunctival mass that was noted to increase in size in a few years' time. Initial examination revealed a 10-mm soft yellowish inferonasal conjunctival mass with a whitish surface. The rest of the examination findings were unremarkable. A computed tomographic scan was done and showed a conjunctival mass that was not connected posteriorly to the orbital fat but had the same density as the orbital fat. Complete surgical excision was performed, and pathologic evaluation of the excised specimen revealed a dermolipoma. The patient was subsequently followed up to monitor for any postoperative complications. Three months following the operation, there was no recurrence of the tumor, and the patient had an excellent cosmetic outcome.

## Introduction

Dermolipoma is the second most common conjunctival mass in children and is the most common epibulbar choristoma [1]. It is a benign mass composed of adipose tissue with keratinized stratified squamous epithelium and is usually located in the superotemporal conjunctival fornix [2-4]. Dermolipomas located in other quadrants are rare. As for the inferonasal quadrant, there have been only three reported cases of inferonasal dermolipoma published in the literature [5-7]. This is not the typical location for dermolipomas, hence emphasizing the unusual presentation of this case.

## Case presentation

This study was conducted in accordance with the tenets of the Declaration of Helsinki and its later amendments. Written informed consent for the publication of identifiable face photos was obtained from the patient’s mother. A nine-year-old boy consulted with our service for the removal of a conjunctival mass. His parents noticed the mass a few years ago, and the lesion had gradually increased in size. The patient had no history of trauma. Past medical history and family history were also unremarkable.

**Figure 1:**
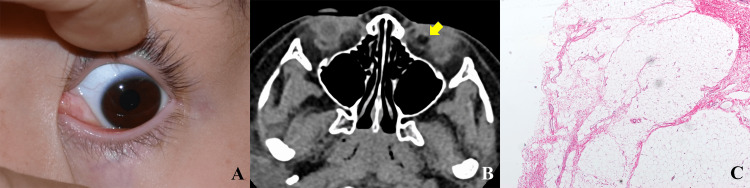
Clinical findings of the patient. A. A soft, yellowish conjunctival mass in the inferonasal quadrant. B. An axial computed tomographic image showing the mass in the left medial canthal area with the same density as the orbital fat, but not connected to the orbital fat (arrow). C. Histologic photograph of the excised specimen showing mature fat cells and fibrous connective tissue.

On initial consultation, his best-corrected visual acuity was 1.2 in both eyes. Refractive errors were insignificant. Intraocular pressures were 19 mmHg in the right eye and 17 mmHg in the left. Extraocular muscle motility was normal. Slit-lamp examination of the left eye revealed a 10-mm soft conjunctival mass in the inferonasal quadrant (Figure 1A). Although the mass was mostly yellowish, a portion of its exposed surface was whitish in color. Computed tomographic (CT) images showed a conjunctival mass with the same density as the orbital fat, but not connected to the orbital fat (Figure 1B). Physical examination did not show any suspicious findings related to Goldenhar syndrome, organoid nevus syndrome (linear nevus of Jadassohn), Treacher-Collins syndrome, colobomas of the eye, osteomas, limbal dermoids, and facial nerve palsy.

The surgery was done under general anesthesia by one of the authors (YT). The mass was completely excised, and the conjunctival defect was closed using 6-0 polyglycolic acid sutures (VSORB; Kono Seisakusho Co., Ltd.; Chiba, Japan). Pathological examinations of the excised specimen showed mature fat cells and fibrous connective tissue covered by stratified squamous epithelium with goblet cells (Figure 1C). The findings of pathological examinations corresponded to the dermolipoma. At the three-month follow-up, vision and extraocular muscle motility were still normal. There was no recurrence of the tumor.

## Discussion

Since dermolipoma is typically found in the superotemporal quadrant, the clinician must have a high level of suspicion in order to diagnose rare cases presenting in an unusual anatomic location. The authors have found only three case reports on dermolipomas presenting inferonasally as protruding conjunctival masses, one of which was found to contain ectopic lacrimal gland tissue [5]. Another case was associated with split hand/foot malformation [6]. The other previous report showed one of 196 dermolipomas (0.5%) located in the inferonasal quadrant [7]. Because of the rarity of dermolipoma cases presenting inferonasally, sex predilection and other risk factors cannot be established as of yet.

Dermolipomas in locations other than the superotemporal and inferonasal quadrants are also rare. A previous report showing 116 ocular dermoids demonstrated that 30% of dermolipomas were found in locations other than the temporal quadrant, but this report did not show further details [8]. Another report including 196 cases of dermolipomas showed those in the inferotemporal, nasal, and superonasal in four, two, and one cases, respectively [7]. Lazzaro et al. presented a case of dermolipoma in the inferotemporal region, which involved the cornea [9]. Maeng et al. and Tripathy and Mittal described two cases of dermolipoma located in the lower eyelid near the inferior fornix [3,10].

Orbital dermolipoma is usually present at birth but may not be visually detected until later in life [3-5]. As the child grows bigger and gains more body fat, the mass can also grow in size. Hence, the preexisting mass becomes more evident to the parent, which can explain the situation in our patient. Furthermore, the whitish appearance on the surface of the mass found in our patient may have been due to chronic exposure in the medial palpebral fissure.

Small and asymptomatic lesions are typically observed. Since the parent found the lesion cosmetically bothersome, surgical treatment was opted for. Dermolipoma may exhibit posterior extension into the orbit without connection to the intraconal orbital fat [4,11], but a CT scan, in this case, revealed no posterior extension. To achieve the best cosmetic outcome, it is important to perform complete surgical excision while preserving the surrounding healthy tissue [3]. The surgeon must also be cautious about damaging the medial rectus, inferior rectus, and inferior oblique muscles. Dermolipoma has a very low potential for malignant transformation, hence periodic monitoring mainly for postoperative complications, such as forniceal shrinkage and strabismus, is recommended [1].

## Conclusions

The authors report a rare case of dermolipoma found in the inferonasal conjunctival fornix, which is an unusual location for this type of tumor. Dermolipoma must, therefore, be considered in a patient presenting with protruding conjunctival mass with a characteristic appearance, regardless of the anatomic location.
